# Conjunctival Squamous Cell Carcinoma Following Intravitreal Injection Through an Unrecognized CIN: A Case Report

**DOI:** 10.1055/a-2772-9119

**Published:** 2026-02-11

**Authors:** Augustina Grigaite, Alexandre Moulin, Ann Schalenbourg

**Affiliations:** 1 Department of Ophthalmology, Jules-Gonin Eye Hospital, Lausanne, Switzerland; 2 Faculty of Biology and Medicine, University of Lausanne, Switzerland; 3 Department of Pathology, Jules-Gonin Eye Hospital, Lausanne, Switzerland

## Background/Introduction

Conjunctival squamous cell carcinoma (SCC) is the most frequent malignant non-pigmented conjunctival tumour, with an estimated incidence of 0.13 to 1.9 per 100,000 1 . It commonly arises from a precancerous lesion called conjunctival intraepithelial neoplasia (CIN). The distinction between SCC and CIN is made histopathologically, the former breaching and invading beyond the basal membrane. Both entities are often jointly referred to as ocular surface squamous neoplasia (OSSN).

Both CIN and SCC usually appear on the interpalpebral conjunctiva and present as a gelatinous or leukoplakic mass, which may have feeder vessels. Its development is associated with UV exposure, smoking, human papilloma virus infection, elder age and a suppressed immune system 2 . The smaller tumours can easily be mistaken for benign lesions, such as a pinguecula or pterygium 1 . SCC does not metastasize unless it is left untreated and invades the orbit and local lymph nodes 3 .

Treatment of OSSN includes excisional biopsy and/or topical chemotherapy, depending on the lesionʼs extent. Adjuvant cryotherapy can be applied to the margins, reducing the risk of a local recurrence 4 . For invasive SCC on the bulbar conjunctiva, plaque therapy destroys malignant cells having penetrated the sclera 5 , thus avoiding intra-ocular dissemination, which manifests itself as an uncontrollable intra-ocular inflammation and nearly always requires enucleation 1 .

To our knowledge, we describe the first case of a conjunctival SCC that appeared following an inadvertent intravitreal injection (IVT) through a pre-existing CIN.

## Case Report

A 92-year-old pseudophakic Caucasian male patient had been receiving IVT injections for an age-related macular degeneration for 3 years. Two weeks after the last injection, he was referred to our hospital because of a conjunctival temporal mass in the same right eye (RE), which had appeared at the injection site and which had not regressed under topical Tobramycin and Dexamethasone treatment.

**Fig. 1:**
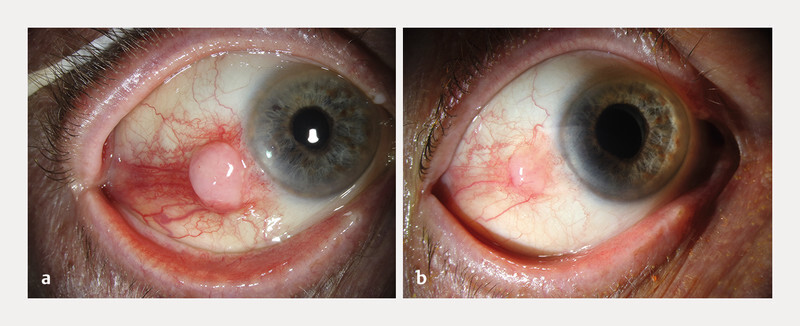
**a** Initial presentation: a non-pigmented, vascularized and immobile conjunctival mass, centred at 4 mm from the temporal-inferior limbus (RE), two weeks following an ipsilateral IVT injection at the same site. **b** Pre-operative presentation, three weeks later: the lesion has partially regressed, presumedly because of reflux after the IVT injection.

Upon examination, a non-pigmented, vascularized and immobile conjunctival mass, centred at 4 mm from the inferior-temporal limbus was observed ( Fig. 1 a ). Its thickness on ultrasound biomicroscopy (UBM) was 2.8 mm. During history, the patientʼs daughter remembered the presence of a whitish, though much smaller and flatter lesion at the same location. A clinical diagnosis of conjunctival SCC was made and a surgical excision, associated with Ruthenium plaque brachytherapy was scheduled.

Three weeks later, during pre-operative examination, the lesion had partially regressed, which was interpreted as due to probable reflux immediately after the injection ( Fig. 1 b ).

The two surgical interventions were uneventful. Ninety Gray (CGE) were delivered at a total depth of 2 mm, in an attempt to destroy malignant cells potentially introduced into the sclera. The conjunctival defect was closed with an autologous graft from the ipsilateral superior bulbar conjunctiva.

**Fig. 2:**
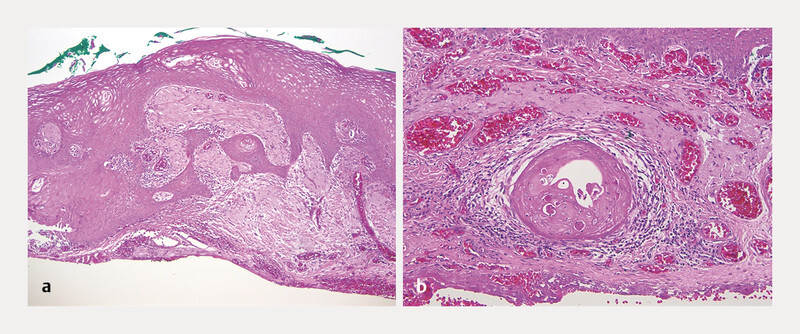
Histopathological analysis: **a** In situ well differentiated carcinoma, with infiltrative tumour strands of SCC. The greyish areas in the stroma correspond to solar elastosis (HE 63×). **b** Micro-invasive SCC within the stroma (HE 126×).

Histopathology confirmed the diagnosis of a predominantly in situ, well-differentiated squamous cell carcinoma containing cells with an enlarged clear cytoplasm. There were also islands of dyskeratotic cells (micro-invasion) within the stroma ( Fig. 2 a and b ).

Upon control examination one year later, no signs of a local recurrence or an intraocular inflammation were observed.

## Discussion

To our knowledge, we report the first case of a conjunctival SCC that appeared following an IVT injection through an unrecognized CIN. To treat it, we opted for a surgical excision and adjuvant Ruthenium plaque brachytherapy, to prevent intra-ocular spread.

Intra-ocular dissemination of OSSN is a rare, but disastrous complication. Kaliki et al. reviewed the clinical features, histopathology and treatment in 23 cases of OSSN with intraocular tumour extension. Over the course of follow-up, extended enucleation (n = 6) or orbital exenteration (n = 17) was required for local tumour control. At a mean follow-up period of 18 months, locoregional lymph node metastasis was seen in two (9%) patients, and one patient died with systemic metastasis. On histopathology, all 23 cases presented ciliary body involvement 3 .

Another key risk factor is a history of prior tumour excision 3 . Shields et al. reported five cases, in all of which an incomplete conjunctival SCC excision had been performed and a recurrent mass presented with signs of intraocular inflammation and secondary glaucoma. All five cases were treated with modified enucleation (n = 3) or exenteration (n = 2). During follow-up, no metastases appeared 6 . Sometimes, the OSSN is mistaken for a pterygium and incompletely excised. Rootman et al. described two cases, followed by a rapid regrowth and signs of intraocular inflammation, requiring enucleation 1 .

A third risk factor, evocative of our case, is the manipulation of malignant tissue during intra-ocular surgeries, e.g. cataract surgery or penetrating keratoplasty, which can inadvertently introduce cancer cells into the eye, subsequently cause uncontrollable ocular inflammation and eventually end up with an enucleation. We found about 10 such cases 1 , 3 , 7 .

The best management of intra-ocular SCC invasion is prevention: following surgical excision of an invasive bulbar SCC, adjuvant brachytherapy is applied, either immediately in case of a clear clinical diagnosis or delayed, once there is a histopathological diagnosis, in order to destroy any intra-scleral malignant cells. Ruthenium-106 plaques have the advantage of delivering high energy beta rays which have a limited tissue penetration depth and donʼt harm deeper structures 8 . This technique was introduced in the 70’s and has ever since been proven as a safe method to achieve good local tumour control 5 , 9 .

We could only identify three case reports of eyes with an intraocular SCC extension, which could be salvaged, with a follow-up of at least one year: one was treated with only iridocyclectomy and two with adjuvant Ruthenium brachytherapy. No recurrence, nor metastases were observed after respectively 3.5-, 2- and 4.5-yearsʼ follow-up 10 .

In conclusion, our case emphasizes the importance of meticulous examination of the bulbar conjunctiva before performing intravitreal injections or any other surgery, to avoid intraocular dissemination of a pre-existing CIN. In case the diagnosis of OSSN is made post-operatively, brachytherapy, e.g. with a Ruthenium plaque, can be used to destroy possible intra-scleral malignant cells and reduce the risk of intra-ocular dissemination.
